# Long noncoding RNA ADEI/miR-93-3p/STAT3 axis promotes Epstein–Barr virus-positive diffuse large B-cell lymphoma progression and immune evasion through regulating the PD-1/PD-L1 checkpoint

**DOI:** 10.1038/s41419-026-08532-4

**Published:** 2026-03-03

**Authors:** Weili Zheng, Guilan Lai, Ziyuan Liao, Jianzhen Shen

**Affiliations:** 1 Department of Lymphoma and Head and Neck Oncology, Clinical Oncology School of Fujian Medical University, Fujian Cancer Hospital, Fuzhou, China; 2 Department of Hematology, the First Affiliated Hospital, Fujian Medical University, Fuzhou, China; 3 Meng Chao Hepatobiliary Hospital Affiliated to Fujian Medical University, Fuzhou, China; 4 Fujian Institute of Hematology, Fujian Medical Center of Hematology, Fujian Provincial Key Laboratory on Hematology; Fujian Medical University Union Hospital, Fuzhou, China

**Keywords:** Cancer microenvironment, B-cell lymphoma

## Abstract

Epstein–Barr virus (EBV) is an important pathogenic factor of lymphoma; EBV+diffuse large B-cell lymphoma (DLBCL) has a worse prognosis with standard chemotherapy than EBV-DLBCL. Long noncoding (Lnc)-RNAs are key regulators of cancer pathways and biomarkers of disease. As natural protective carriers of noncoding RNAs, exosomes can stably transmit signals during tumor development. We explored the role of exosomal lncRNAs in the occurrence and development of EBV-related DLBCL. In this study, we identified a novel lncRNA lncADEI, which was upregulated in EBV + DLBCL and was positively correlated with DLBCL cell proliferation and clonogenesis. LncADEI positively regulated STAT3 via miR-93-3P, and STAT3 transcriptionally activated programmed death ligand-1 to promote immune evasion of DLBCL cells. LncADEI could be transferred by exosomes and promote the proliferation and immune evasion of DLBCL. LncADEI was highly expressed in serum exosomes from EBV + DLBCL patients and was associated with worse clinicopathological features. In conclusion, lncADEI participated in the progression and immune evasion of EBV + DLBCL and was differentially expressed in serumal exosomes. LncADEI may be a promising strategy for treating EBV-associated lymphoid malignancies.

## Introduction

Diffuse large B-cell lymphoma (DLBCL) is the most common B-cell malignancy, accounting for 25–35% of all non-Hodgkin lymphomas [1]. Although chemotherapy with R-CHOP has led to significant advances in DLBCL treatment, ~40% of patients relapse or do not respond to treatment [2, 3]. Epstein–Barr virus-positive DLBCL (EBV + DLBCL) has a poor prognosis when treated with standard chemotherapy [4]. Therefore, there is an urgent need to further study the role and mechanism of EBV infection in the occurrence and development of DLBCL and to identify new therapeutic targets.

LncRNAs are transcriptions with >200 nucleotides in length and are mainly characterized by a lack of protein-coding functions [5]. Increasing evidence has demonstrated that lncRNAs can be used as key regulators of cancer pathways and disease biomarkers [6]. To identify several differentially expressed lncRNAs in DLBCL has become an increasing interest for researchers. For example, specific lncRNAs, such as HOTAIR, SNHG15, and H19, could offer potential for DLBCL staging and patient prognosis [7]. LINC01949 suppressed rituximab resistance in DLBCL via H3K27me3-mediated ONECUT2 silencing [8]. Lnc SMAD5-AS1 promotes the proliferation of DLBCL cells and leads to tumor progression by increasing the expression of APC and inactivating the Wnt/β-catenin pathway through sponge miR-135b-5p [9]. Nevertheless, the role of lncRNAs in the occurrence and development of EBV-related DLBCL requires further exploration.

Programmed death ligand-1 (PD-L1), a B7 family ligand, interacts with its receptor programmed death-1 (PD-1) to inhibit the lifespan of cytotoxic CD8+ T cells, resulting in immune escape [10]. Therefore, blocking the PD-1/PD-L1 checkpoint with antibodies has become a therapeutic strategy for various aggressive tumors, including lymphoma, but the effective rate is only ~20% [11]. Hence, the identification of relevant targets that regulate the PD-1/PD-L1 pathway is helpful in improving the clinical efficacy of DLBCL immunotherapy.

Exosomes are extracellular microvesicles with a diameter of 40–160 nm that are secreted into body fluids by different cell types [12]. Exosomes can contain various nucleic acids, proteins, and other components such as lncRNA, microRNA, circular RNA, and other nonencoding (nc)-RNA [13]. Exosomes from host cells can be taken up by recipient cells and play biological roles in neighboring or distant recipient cells. Therefore, exosomes can play a variety of functions in the adaptive immune response and malignant tumorigenesis [14]. Additionally, studies have shown that EBV-infected B lymphoma cells secrete an abnormally high number of exosomes [15]. Moreover, these exosomes can affect the function of immune cells in the tumor microenvironment, thereby participating in the occurrence and development of B-cell lymphoma [16].

In the present study, we performed lncRNA microarray analysis of DLBCL exosomes according to EBV infection status to explore the role of exosomal lncRNAs in the occurrence, development and immune evasion of EBV-related DLBCL.

## Methods

### Ethics statements

The study was approved by the Ethics Committee of Fujian Cancer Hospital, and informed consent was obtained from all participants in accordance with the Declaration of Helsinki.

### Cell lines

The human B-cell lymphoma cell line (SU-DHL-2 and DB) and EBV-producing marmoset B-cell line B95-8 were kindly provided by the Stem Cell Bank, Chinese Academy of Sciences. Cells were cultured in RPMI-1640 (Gibco, China) in a humidified atmosphere of 95% air and 5% CO_2_ at 37 °C. Human renal epithelial cells (HEK-293T) were gifted by the Fujian Institute of Hematology and were grown in DMEM. All cell lines were identified by STR profiling. EBV-infected cells were established as previously described [17].

### Patient samples

In total, 47 serum samples with newly diagnosed DLBCL were obtained from the Fujian Cancer Hospital. DLBCL cases were confirmed by pathological examination of lymph node biopsy or lymphadenectomy, according to the 2017 World Health Organization Classification of Lymphoid Neoplasms. Informed consent was obtained from all subjects.

### Statistical analysis

All experiments were performed independently with at least three biological replicates. Data are presented as mean ± standard deviation. Statistical analyses were performed using GraphPad Prism 8 (GraphPad, San Diego, USA) and SPSS 22.0 software (IBM Corp.). Differences between two groups were analyzed using the independent sample *t*-test, and differences between multiple groups were analyzed using one-way analysis of variance. Patients’ clinical characteristics were analyzed using the χ^2^ test. Statistical significance was defined as *P* < 0.05.

## Results

### EBV infection promoted DLBCL progression and immune evasion through regulating the PD-1/PD-L1 checkpoint

**Fig. 1:**
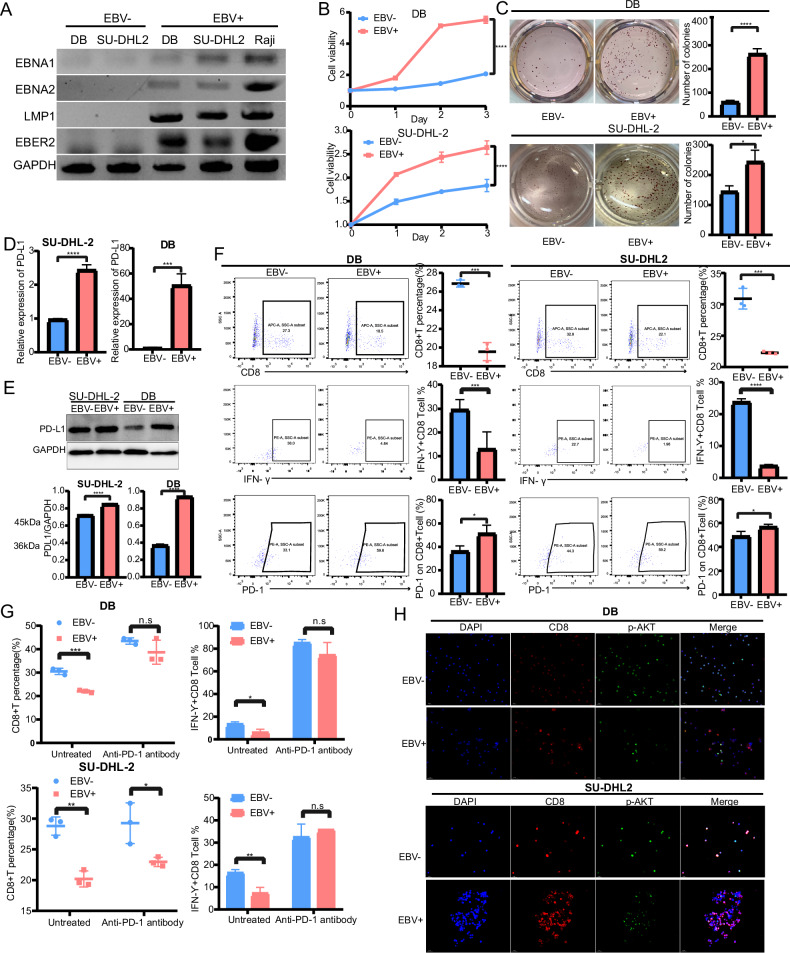
EBV infection promoted DLBCL progression and immune evasion by regulating the PD-1/PD-L1 checkpoint. **A** RT-PCR reveals that *EBNA1*, *EBNA2*, *LMP1*, and *EBER2* were detected only in EBV + DLBCL cell lines. The EBV-positive Raji cell line was used as a positive control. **B** CCK-8 assays show the cell proliferation ability of EBV +/− DLBCL cell lines. **C** The quantification and representative images of EBV +/− DLBCL cells analyzed by colony formation assays. **D** The expression of PD-L1 in EBV +/− DLBCL cells was detected by qRT-PCR. **E** Western blotting and densitometric analyses show PD-L1 protein levels in EBV +/− DLBCL cells. **F** The CD8+ T cell percentage, expression of cytotoxicity (IFN-γ) and exhaustion (PD-1) were detected using flow cytometry analysis when co-cultured with EBV +/− DLBCL. **G** CD8+ T cells percentage and cytotoxicity were detected upon treatment with anti-PD-1 antibody in the coculture system of EBV +/− DLBCL. **H** Immunofluorescence results show that the expression of p-AKT was decreased in CD8+ T cells co-cultured with EBV + DLBCL compared with EBV-DLBCL (magnification ×400). All data represented mean ± s.d. from three independent experiments. NS: *P* > 0.05; *P < 0.05; **P < 0.01; ***P < 0.001; ****P < 0.0001.

To explore the effect of the EBV virus on DLBCL, we constructed DLBCL cell lines stably infected with EBV virus (EBV + DB, EBV + SU-DHL-2) and verified the expression of EBV-associated viral genes *EBER2*, *EBNA1*, *EBNA2*, and *LMP1* by reverse transcription PCR (Fig. 1A). Compared with EBV-DLBCL, EBV enhanced DLBCL cell proliferation and clonogenesis (Fig. 1B, C). We further explored the effect of EBV infection on immune escape in DLBCL. The results demonstrated that EBV + DLBCL cells had higher PD-L1 expression at the mRNA and protein levels according to qRT-PCR and Western blot analyses (Fig. 1D, E). To simulate the tumor microenvironment, DLBCL cells were co-cultured with immune cells. Flow cytometry analysis showed that EBV + DLBCL significantly reduced the proportion of CD8+ T cells, attenuated the cytotoxicity of CD8+ T cells (IFN- γ) and increased PD-1 expression in CD8+ T cells (Fig. 1F). Furthermore, the effect of EBV + DLBCL cells on CD8+ T cell percentage and activity (IFN- γ) was abrogated by anti-PD-L1 antibody in the co-culture systems (Fig. 1G). A previous study reported that exhausted CD8+ T cells in chronic EBV infection would continue to activate the PD-1/PD-L1 pathway, which could affect downstream Akt inactivation, leading to further T cell dysfunction and apoptosis [18]. Indeed, p-AKT expression in CD8+ T cells was decreased in the EBV + DLBCL co-culture system compared to that in EBV-DLBCL cells (Fig. 1H). Together, EBV infection aggravated DLBCL progression and induced PD-1/PD-L1 activation in tumor microenvironment.

### LncADEI was upregulated in EBV + DLBCL, and promoted DLBCL progression and immune evasion through regulating the PD-1/PD-L1 checkpoint

**Fig. 2:**
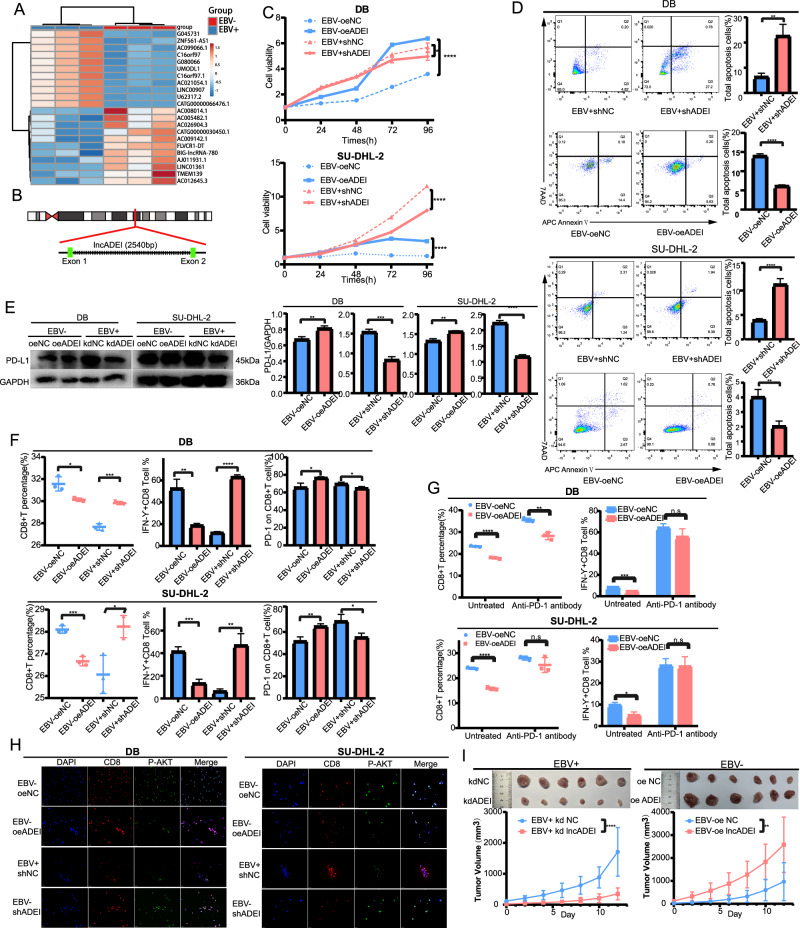
LncADEI is upregulated in EBV + DLBCL and promotes DLBCL progression and immune evasion. **A** Heatmap displaying the most significantly different lncRNAs; red and blue represent upregulated and downregulated lncRNAs, respectively. **B** Genomic location and mode of lncADEI. **C** Cell proliferation ability of lncADEI^sh^EBV + DLBCL, lncADEI^oe^EBV-DLBCL, and control cells detected using CCK-8 assays. **D** The effect of lncADEI on apoptosis of EBV +/− DLBCL cells was measured through flow cytometry analysis. The representative images are on the left, and quantifications are on the right. **E** Western blot and densitometric analyses showing PD-L1 protein levels in transfected cells. **F** The CD8+ T cell percentage, IFN- γ and PD-1 expression were detected using flow cytometry analysis when co-cultured with transfected DLBCL cells. **G** CD8+ T cells percentage and cytotoxicity were detected upon treatment with anti-PD-1 antibody in the co-culture system of lncADEI^oe^EBV-DLBCL compared with control groups. **H** Immunofluorescence results show that the p-AKT expression decreased in CD8+ T cells co-cultured with lncADEI^oe^EBV-DLBCL and increased in CD8+ T cells co-cultured with lncADEI^sh^EBV + DLBCL compared to the control group, respectively (magnification ×400). **I** Pictures of xenografts and quantification of the volume of tumors in lncADEI-knockdown EBV + DB cells, lncADEI-overexpressing EBV-DB cells and vector groups. LncADEI promotes DLBCL tumor growth in vivo. All data represented mean ± s.d. from three independent experiments. *P < 0.05; **P < 0.01; ***P < 0.001; ****P < 0.0001.

First, a lncRNA microarray was used to compare the lncRNA expression profiles between EBV + DB and EBV-DB cell supernatant exosomes. In total, 336 lncRNAs were found to be significantly upregulated and 364 lncRNAs were downregulated in EBV + DLBCL (Supplementary Fig. S1A) (log2 fold change ˃1.5, *P* < 0.05). Heatmaps (Fig. 2A) showed partial differential lncRNAs between EBV +/− DLBCL cell exosomes. Six of the most differentially expressed lncRNAs were validated by qRT-PCR using EBV +/− DB cell lines (Supplementary Fig. S1B). Subsequently, the three significantly different lncRNAs were verified in EBV +/− SU-DHL-2 cells and exosomes (Supplementary Fig. S1C). Ultimately, we focused on an uncharacterized lncRNA (T198242) and named lncADEI (lncRNA Activated in DLBCL with EBV infection). LncADEI is located on human chromosome 2 q14.3 and consists of two exons with a total length of 2540 nt (Fig. 2B). To investigate the function of lncADEI in EBV-infected DLBCL, we established stable lncADEI-knockdown EBV + DLBCL cells, overexpressed EBV-DLBCL cells, and verified the results by q-PCR (Supplementary Fig. S1D). Cell proliferation (Fig. 2C) and clonogenesis (Supplementary Fig. S1E) were enhanced by overexpression of lncADEI in EBV-DLBCL cells and inhibited by knockdown in EBV + DLBCL cells, compared with the control groups. Moreover, apoptosis assays showed that lncADEI knockdown significantly increased the apoptosis of EBV + DLBCL cells compared to the control group. In contrast, lncADEI overexpression decreased the percentage of apoptosis (Fig. 2D). Additionally, we examined the effect of lncADEI on immune escape in DLBCL. Western blotting and q-PCR results showed that silencing lncADEI reduced PD-L1 mRNA (Supplementary Fig. S1F) and protein expression (Fig. 2E). Flow cytometry revealed a decrease in the proportion and cytotoxicity of CD8+ T cells and an increase in PD-1 expression in the EBV-DLBCL cells overexpressing lncADEI (Fig. 2F). However, the effect of lncADEI overexpression on CD8+ T cell percentage and function was eliminated by anti-PD-1 antibody in the co-culture systems (Fig. 2G). It indicated that the increase in CD8+ T cells cytotoxicity following anti-PD-1 treatment was more pronounced in the lncADEI overexpression group than in the vector group, highlighting the enhanced responsiveness to PD-1 blockade induced by lncADEI overexpression. Furthermore, p-AKT expression in CD8+ T cells was upregulated in the co-culture system of lncADEI-knockdown EBV+cells and downregulated in that of lncADEI-overexpressing EBV-cells (Fig. 2H). Finally, the effect of lncADEI on DLBCL tumor growth was verified through animal experiments. Murine xenograft models were established with stable lncADEI-knockdown EBV + DB cells, lncADEI-overexpressing EBV-DB cells and vector groups. Consistent with in vitro data, low expression of lncADEI significantly reduced the tumor volume in EBV + DLBCL compared to that in the control group. In contrast, lncADEI overexpression increased the tumor volume (Fig. 2I). Collectively, lncADEI was highly expressed in EBV + DLBCL cells and significantly affected proliferation, apoptosis, and immune escape via PD-1/PD-L1 activation in TME.

### LncADEI interacted with miR-93-3p in DLBCL cells

**Fig. 3:**
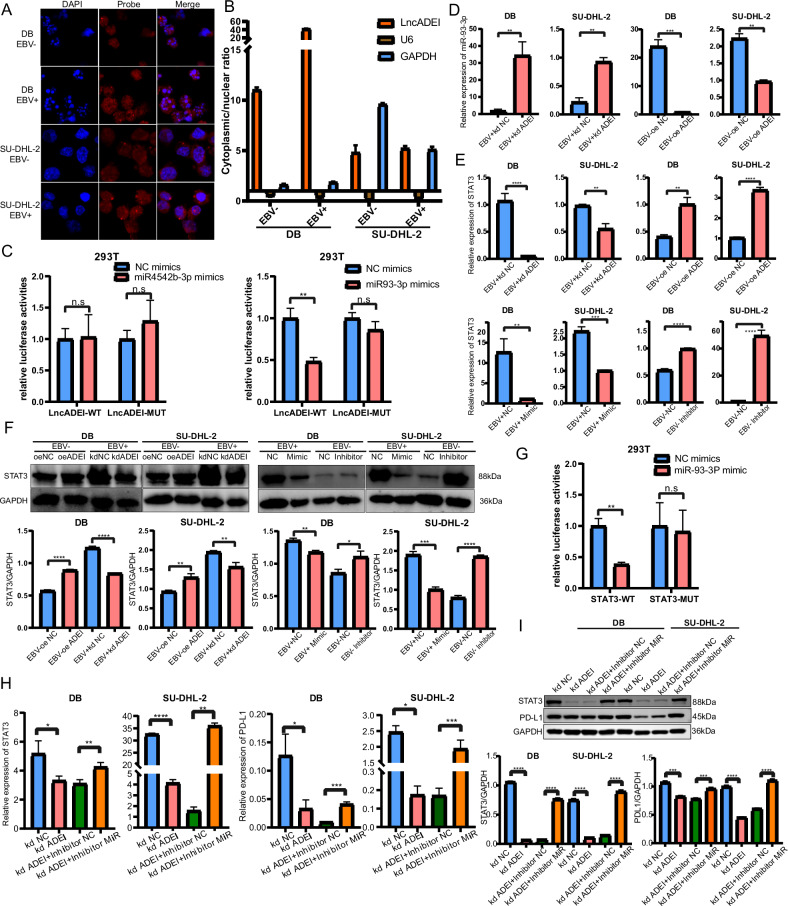
LncADEI interacts with miR-93-3p in DLBCL cells. **A** FISH localization of lncADEI was observed by confocal laser microscopy. DAPI stains the nucleus, and red Cy3 dye labels the lncADEI FISH probe (magnification ×600). **B** The qRT-PCR analysis was used to analyze the expression of lncADEI in the cytoplasm and nucleus. U6 and GAPDH acted as nuclear and cytoplasmic reference genes, respectively. **C** MiR-93-3p reduces the lncADEI luciferase reporter, and this effect disappears after mutation; miR-4542b-3p does not significantly alter the luciferase reporter. **D** The q-PCR results show that miR-93-3p was highly expressed in lncADEI^sh^EBV + DLBCL and lowly expressed in lncADEI^oe^EBV-DLBCL compared to NC cells, respectively. **E** The q-PCR analysis detects the mRNA expression levels of STAT3 in cells overexpressing or knocking down lncADEI/miR-93-3p, respectively. **F** Western blotting and densitometric analyses showing STAT3 protein levels in overexpression or knockdown of lncADEI/miR-93-3p cells (Mimic and inhibitor referred to overexpression of miR-93-3p and inhibition of miR-93-3p, respectively). **G** MiR-93-3p reduces the STAT3 luciferase reporter, and this effect disappears after mutation. **H** The q-PCR analysis detects the mRNA expression levels of STAT3 and PD-L1 in EBV + DLBCL cells after co-transfection with kdADEI and miR-93-3P inhibitor vectors. **I** Western blotting and densitometric analyses showing STAT3 and PD-L1 in EBV + DLBCL cells after co-transfection with kdADEI and miR-93-3P inhibitor vectors. WT wild type, Mut mutation, FISH fluorescence in situ hybridization, DAPI 4’,6-diamidino-2-phenylindole. All data represented mean ± s.d. from three independent experiments. NS: *P* > 0.05; *P < 0.05; **P < 0.01; ***P < 0.001; ****P < 0.0001.

Next, we explored the molecular mechanism of lncADEI in DLBCL. The Cy3-labeled lncADEI probe was designed to detect intracellular distribution using FISH. The results showed that lncADEI was mainly distributed in the cytoplasm (Fig. 3A). We also isolated the cytoplasm from the nucleus and detected the subcellular localization of lncADEI using q-PCR. Similar to previous FISH results, lncADEI was mainly expressed in the cytoplasm (Fig. 3B). Therefore, we explored whether lncADEI interacted with microRNAs in DLBCL. We selected five microRNAs that were most related to lncADEI from the prediction results of Starbase3.0. The q-PCR analysis revealed that miR-93-3p and miR-4542B-5p were significantly downregulated in EBV + DLBCL cells compared with EBV-DLBCL cells, suggesting that these two microRNAs were associated with EBV infection in DLBCL (Supplementary Fig. S2A). To study the detailed interaction between microRNAs and lncADEI, we mutated the predicted miR-93-3p and miR-4542B-5p sites on lncADEI for luciferase reporter assay (Supplementary Fig. S2B). The luciferase activity of (wild-type) WT lncADEI was significantly decreased by overexpression of miR-93-3p, but not miR-4542b-5p. Moreover, miR-93-3p overexpression failed to affect the luciferase activity of mutant (Mut) lncADEI (Fig. 3C). This finding suggests that lncADEI interacted with miR-93-3p instead of miR-4542B-5p at the predicted binding site. Moreover, we detected a regulatory relationship between miR-93-3p and lncADEI in DLBCL cells. The q-PCR analysis showed that miR-93-3p levels increased in EBV + DLBCL cells with lncADEI knockdown. Conversely, overexpression of lncADEI resulted in a low level of miR-93-3p in EBV-DLBCL (Fig. 3D). Together, lncADEI was mainly localized in the cytoplasm and negatively interacted with miR-93-3p in DLBCL cells.

### LncADEI regulated STAT3 expression via miR-93-3p

We predicted potential target genes of miR-93-3p using TargetScan. Interestingly, we found that STAT3 was a potential target of miR-93-3p. EBV infection of B lymphocytes activates the transcription factor STAT3 [19], and EBV-encoded miR-BART5-5p upregulates PD-L1 by regulating pSTAT3 [20]. Therefore, we deduced that lncADEI/miR-93-3p regulates STAT3 to alter PD-1/PD-L1 expression in DLBCL. To determine whether lncADEI or miR-93-3p could regulate STAT3 expression, we constructed and validated EBV + DLBCL cells overexpressing miR-93-3p (Mimic EBV + DLBCL) and EBV-DLBCL with miR-93-3p knockdown (Inhibitor EBV-DLBCL) (Supplementary Fig. S2C). Then, we detected STAT3 expression in lncADEI or miR-93-3p knockdown and overexpressing DLBCL cells using q-PCR. The mRNA and protein expression levels of STAT3 were significantly decreased by lncADEI knockdown or miR-93-3p overexpression in EBV + DLBCL cells compared with those in the control groups (Fig. 3E, F). STAT3 levels in the lncADEI overexpression or miR-93-3p inhibition groups were higher than those in the EBV-DLBCL cells. The interacting sequences of STAT3 for miR-93-3p and the Mut sequences are presented in Supplementary Fig. S2D. Furthermore, miR-93-3p overexpression decreased the luciferase activity of WT STAT3, but not Mut STAT3 (Fig. 3G). To further verify if lncADEI could regulate STAT3 level via miR-93-3p, we transfected the lncADEI-knockdown EBV+cells with miR-93-3p inhibitor (Supplementary Fig. S2E). Results showed lncADEI knockdown decreased STAT3 and PD-L1 mRNA and protein expression levels, while a low level of miR-93-3p rescued the decreased STAT3 and PD-L1 levels induced by lncADEI silence (Fig. 3H, I). Silencing lncADEI inhibited the proliferation of EBV + DLBCL cells, while miR-93-3p inhibitor rescued the effects of lncADEI silence (Supplementary Fig. S2F). These results suggested that STAT3 may be positively regulated by lncADEI via miR-93-3p.

### LncADEI promoted DLBCL progression and PD-L1 expression through STAT3

**Fig. 4:**
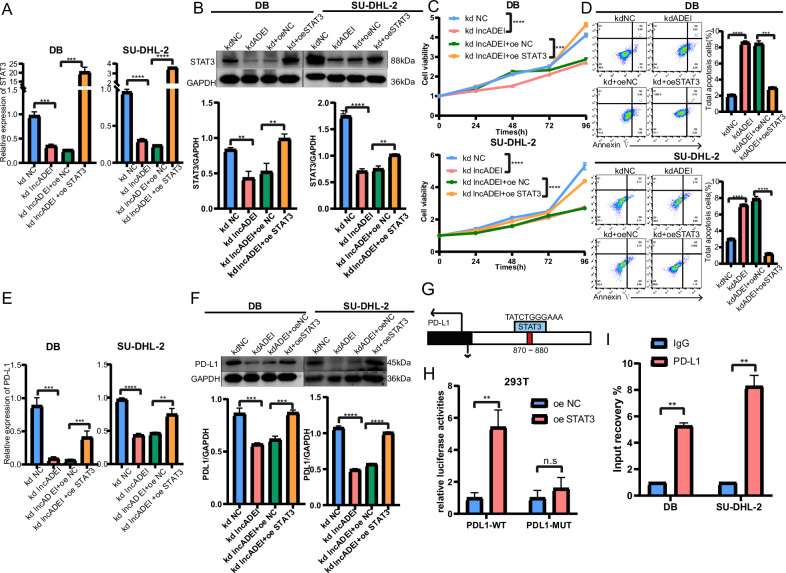
LncADEI promotes DLBCL progression and PD-L1 expression through STAT3. STAT3 expression levels in EBV + DLBCL cells transfected with sh-NC, sh-lncADEI, sh-lncADEI+oe-NC, and sh-lncADEI+oe-STAT3 viruses, respectively. **A**, **B** STAT3 mRNA levels in transfected EBV + DLBCL cells determined using qRT-PCR and Western blotting analyses. **C** Cell proliferation ability of transfected EBV + DLBCL cells. **D** The apoptosis of EBV + DLBCL cells after transfection was detected by flow cytometry. The representative images are on the left, and quantifications are on the right. **E**, **F** PD-L1 mRNA and protein levels in transfected EBV + DLBCL cells determined using q-PCR and Western blotting analyses. **G** Predicted STAT3 binding site in the PD-L1 promoter was acquired using bioinformatics analysis. **H** The dual-luciferase reporter gene assay was performed to show that STAT3 regulated PD-L1 as a transcription factor. **I** ChIP assay shows that STAT3 was bound to the PD-L1 promoter. All data represented mean ± s.d. from three independent experiments. NS: *P* > 0.05; **P < 0.01; ***P < 0.001; ****P < 0.0001.

Further rescue assays were performed to determine whether STAT3 required lncADEI regulation for DLBCL progression and immune escape. First, we confirmed that the mRNA and protein expression levels of STAT3 were reduced by lncADEI depletion and were recovered by STAT3 overexpression in EBV + DLBCL cells (Fig. 4A, B). Silencing lncADEI inhibited the proliferation (Fig. 4C) and clonogenesis (Supplementary Fig. S2G) of EBV + DLBCL cells, while forced expression of STAT3 abolished the inhibitory effects of lncADEI silence. Moreover, inhibiting lncADEI promoted apoptosis of EBV + DLBCL cells, but this effect was countervailed by enhancing the expression of STAT3 (Fig. 4D). Additionally, knockdown of lncADEI reduced the mRNA and protein levels of PD-L1 in DLBCL, while overexpression of STAT3 rescued the low expression of PD-L1 caused by lncADEI silence (Fig. 4E, F). These results suggest that lncADEI promotes DLBCL progression and immune escape by enhancing PD-L1 expression via STAT3. Subsequently, we investigated the regulation of PD-L1 expression by STAT3 in detail. STAT3 is a well-known transcription factor that regulates transactivation of target genes. Therefore, we explored whether STAT3 regulates PD-L1 expression in DLBCL at the transcriptional level. We identified the binding site in the PD-L1 promoter for STAT3 via the JASPAR tool (Fig. 4G). The luciferase reporter assay showed that the PD-L1 promoter reporter was increased by overexpression of STAT3, and this effect could be reversed by mutating the binding site (Fig. 4H). Additionally, the chromatin immunoprecipitation (CHIP) assay demonstrated that the DNA fragments of the PD-L1 promoter were enriched in the precipitates of the STAT3 antibody (Fig. 4I). These data suggest that STAT3 transcriptionally upregulates PD-L1 expression in DLBCL cells. In summary, lncADEI promoted DLBCL progression through STAT3, and STAT3 transcription upregulated PD-L1, thereby promoting DLBCL immune escape.

### LncADEI participated in EBV +/− cell communication via exosomes

**Fig. 5:**
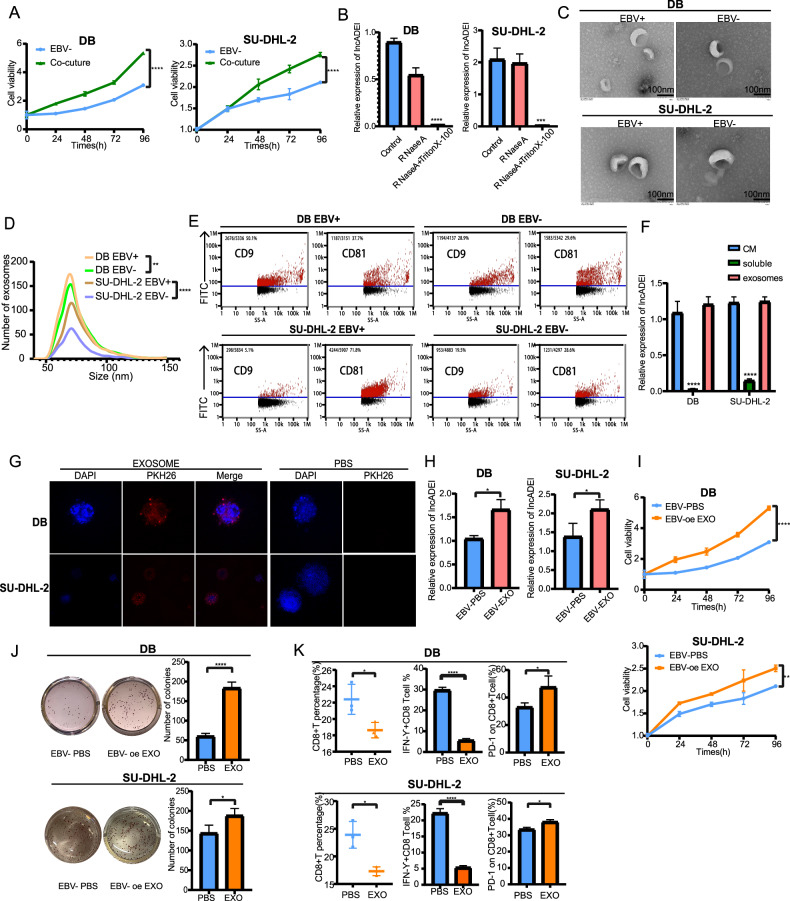
LncADEI participates in EBV +/− cells communication via exosomes. **A** CCK-8 assays show that co-culture with EBV + DLBCL cells enhanced EBV-DLBCL cell proliferation. **B** Q-PCR analysis of lncADEI in the CM of EBV + DLBCL cells treated with RNase (2 mg/ml) alone or combined with Triton X-100 (0.1%) for 20 min (*n* = 3). **C** EBV +/− DLBCL exosomes identified by transmission electron microscopy (scale bar: 100 nm). **D** The size distribution and the number of EBV +/− DLBCL exosomes detected by nanoparticle tracking analysis (NTA). **E** Nanoflow analysis detects the expression of exosome marker proteins CD9 and CD81 in EBV +/− DLBCL exosomes. **F** Q-PCR analysis of lncADEI in exosomes, soluble fraction of CM (soluble) and whole CM derived from EBV + DLBCL cells. **G** Confocal microscopy shows that EBV-DLBCL cells took up EBV + DLBCL exosomes labeled with PKH26 fluorescence, but no fluorescence signal was observed in the PBS group. **H** LncADEI expression of EBV-DLBCL cells co-cultured with EBV + DLBCL exosomes is detected by q-PCR. **I** Cell proliferation ability of EBV-DLBCL co-cultured with lncADEI^oe^EBV-DLBCL exosomes is detected by CCK-8 assays. **J** The quantification and representative images of EBV-DLBCL co-cultured with lncADEI^oe^EBV-DLBCL exosomes analyzed by colony formation assays. **K** After co-culture with lncADEI^oe^EBV-DLBCL exosomes, EBV-DLBCL alters the percentage, IFN- γ, and PD-1 expression of CD8+ T cells. All data represented mean ± s.d. from three independent experiments. *P < 0.05; **P < 0.01; ***P < 0.001; ****P < 0.0001.

To determine whether EBV + DLBCL cells affect the function of EBV-cells, we co-cultured EBV-cells with EBV+cells. The results showed that the proliferation of EBV-DLBCL cells increased after co-culturing with EBV+ cells (Fig. 5A). We next investigated the existing pattern of extracellular lncADEI. The levels of lncADEI in CM were unchanged upon RNase treatment but significantly decreased when treated with RNase and Triton X-100 simultaneously (Fig. 5B), indicating that extracellular lncADEI was mainly wrapped by membrane instead of being directly released. Studies have shown that exosomes play an important role in cell communication; therefore, we wondered whether EBV+ cells could affect EBV-DLBCL cells through exosome transfer of lncADEI. Therefore, we extracted exosomes from the supernatant of EBV +/− DLBCL cells via ultracentrifugation. The extracted exosomes from EBV + /- DLBCL cells exhibited similar typical membrane structures and sizes, as observed by TEM (Fig. 5C). Nanoparticle tracker analysis (NTA) showed that the diameter distribution of exosomes in EBV +/− DLBCL cells was between 50 and 100 nm, and the concentration of exosomes in the supernatant of EBV + DLBCL cells was higher than that in EBV-DLBCL cells (Fig. 5D). The expression of the exosome marker proteins CD9 and CD81 was detected by nanoflow cytometry (Fig. 5E). Intriguingly, lncADEI levels in exosomes were almost equal to that in whole CM (Fig. 5F), indicating that exosome was the main carrier for extracellular lncADEI. To confirm that EBV + DLBCL exosomes could be taken up by receptor cells, EBV-DLBCL cells were co-cultured with EBV + DLBCL exosomes labeled with PKH67, and fluorescent-labeled exosomes were observed in the receptor cells after 48 hours of incubation using a fluorescence microscope (Fig. 5G). These results indicated that EBV + DLBCL exosomes could be taken up by EBV-DLBCL cells. Additionally, lncADEI expression levels increased in EBV-DLBCL cells after co-culture with EBV + DLBCL exosomes by q-PCR analysis (Fig. 5H). We further investigated the roles of exosomal lncADEI in cell proliferation, clonogenesis, and CD8+ T cell activity in co-culture with lncADEI-oe exosomes in EBV-DLBCL cells. The results showed that lncADEI-oe exosomes increased the proliferation and clonogenic ability of EBV-DLBCL cells (Fig. 5I, J). Ultimately, lncADEI-oe exosomes decreased the proportion and cytotoxicity of CD8+ T cells and increased the expression of PD-1 on CD8+ T cells in the co-culture system (Fig. 5K). Overall, these results indicated that EBV + DLBCL exosomes contained lncADEI can be taken up by EBV-DLBCL cells to participate in cell-cell communication, further affect the proliferation of DLBCL cells and interaction with CD8+ T cells.

### Exosomal lncADEI was significantly elevated in EBV + DLBCL patients and indicated lymphoma progression

**Fig. 6:**
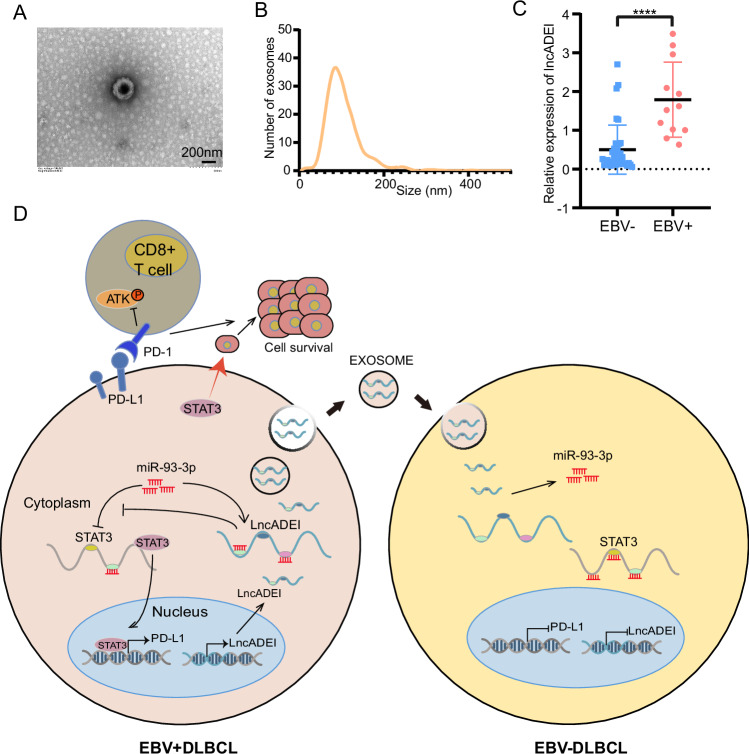
Exosomal lncADEI is significantly elevated in EBV + DLBCL patients. **A** Identification of DLBCL serum exosomes by transmission electron microscopy (scale bar: 200 nm). **B** The size distribution and number of DLBCL serum exosomes detected by nanoparticle tracker analysis (NTA). **C** LncADEI expression in exosomes from DLBCL serum detected by q-PCR. **D** Schematic model showed the role of exosomal lncADEI in EBV-associated DLBCL. ****P < 0.0001.

To further confirm whether exosomal lncADEI is associated with EBV infection of DLBCL, we evaluated the expression level of lncADEI in plasma exosome samples from 47 DLBCL patients. Exosomes were isolated from the serum of 12 EBER-positive DLBCL patients and 35 EBV-DLBCL patients, and identified by TEM (Fig. 6A). NTA also showed a concentration of 9.5 × 10^9^/mL, and the size distribution of the exosomes was within 200 nm (Fig. 6B). LncADEI was highly expressed in exosomes from the serum of EBV + DLBCL patients compared to EBV-DLBCL patients, as detected by q-PCR, suggesting that exosomal lncADEI expression was related to EBV infection in DLBCL (Fig. 6C).

To evaluate the clinical relevance of exosomal lncADEI expression in DLBCL, the median value of relative exosomal lncADEI expression was defined as the cut-off value to divide DLBCL patients into high- and low-lncADEI expression groups. The result showed that exosomal lncADEI expression was positively correlated with poor performance status (Ann-Arbor stage III/IV, *P* = 0.017), pathological type (non-germinal center B, *P* = 0.011), high-risk international prognostic index (3–5, *P* = 0.002), and elevated serum lactate dehydrogenase (LDH) (*P* = 0.005) (Table 1). These results indicated that exosomal lncADEI has the potential to indicate EBV infection and progression of DLBCL.

**Table 1:** Correlation analysis between plasma exosome lncADEI and clinical characteristics in DLBCL patients.

Characteristics	LncADEI		*P* value
	Low expression	High expression	
Age			0.485
≤60	12	15	
>60	11	9	
Gender			0.672
Male	11	13	
Female	12	11	
Ann-Arbor stage			0.017^*^
Ⅰ/Ⅱ	10	3	
Ⅲ/Ⅳ	13	21	
IPI score			0.002^*^
0-2	17	7	
3–5	6	17	
Hans			0.011^*^
GCB	13	5	
Non-GCB	10	19	
LDH			0.005^*^
Normal	15	6	
Elevated	8	18	
CR			0.114
No	9	15	
Yes	14	9	

*IPI* International Prognostic Index, *GCB* Germinal center B-cell, *LDH* Lactic dehydrogenase, *CR* Complete remission.

^*^P < 0.05.

## Discussion

EBV + DLBCL is an aggressive form of non-Hodgkin lymphoma with inferior clinical outcomes, poor response to conventional immunochemotherapy, and frequent recurrence [21]. Similarly, we found that EBV + DLBCL cells had stronger cell proliferation and clonogenic abilities than the control. Moreover, it revealed that EBV could upregulate PD-L1 expression in DLBCL cells and then activate the PD-1/PD-L1 pathway to change the activity of CD8+ T cells in the tumor microenvironment and promote immune escape. Previous studies have shown that EBV infection is significantly correlated with strong PD-L1 expression in DLBCL tumor tissues, and EBV-encoded EBNA2 protein can enhance PD-L1 expression in B-cell lymphomas by downregulating miR-34a [22, 23]. Boussiotis [24] revealed that PD-1 binds to its ligand to recruits the phosphorylases SHP-1 and SHP-2, activates immune receptor tyrosine inhibitory motifs, and induces T cell dysfunction and even apoptosis by inhibiting PI3K-Akt and other signals. Herein, we demonstrated that EBV infection reduced the ratio, cytotoxicity of CD8+ T cells and p-AKT in CD8+ T cells, indicating that EBV infection may induce CD8+ T cell exhaustion by inhibiting p-Akt expression by activating the PD-1/PD-L1 pathway.

LncRNAs are a major type of non-coding RNA, with modest sequence conservation and are highly tissue-specific, and thus have remarkable potential to act as effective biomarkers. However, the role of lncRNAs in EBV-associated tumors has rarely been reported [25]. In the present study, lncADEI was identified as a novel lncRNA that is significantly upregulated in EBV + DLBCL and was positively related to EBV infection, DLBCL progression, and immune escape, suggesting a potential new molecular target for EBV + DLBCL. In vitro and in vivo studies further revealed that lncADEI promoted EBV + DLBCL progression by inducing the proliferation and inhibiting the apoptosis of lymphoma cells. Recently, several studies have implicated the role of lncRNAs in tumor development through proliferation, epithelial-mesenchymal transition, invasion, and migration [26, 27]. Several lncRNAs have also been demonstrated to control the tumor microenvironment by influencing the number, type, and activity of immune cell populations [28]. Here, we demonstrated that lncADEI increases PD-L1 expression in DLBCL cells and promotes DLBCL immune escape by depleting CD8+ T cells in the microenvironment. However, since lncADEI has no homologous mouse sequence, we could not verify the effect of lncADEI on DLBCL immune escape in vivo.

LncRNAs have been reported to act as competing endogenous RNAs interfering with microRNA-mRNA interactions, thereby exerting regulatory functions at the post-transcriptional level [29]. Mechanistically, we revealed the role of lncADEI as a microRNA sponge in DLBCL. First, our findings showed that lncADEI was mainly expressed in the cytoplasm, suggesting that lncADEI might affect the function of DLBCL cells at the post-transcriptional level. Bioinformatic analysis revealed multiple microRNA-binding sites in the lncADEI sequence. Among the candidate microRNAs targeted by lncADEI, miR-93-3p was overtly downregulated in EBV + DLBCL and presented strong affinity with lncADEI, indicating that lncADEI functions in DLBCL mainly through miR-93-3p. Furthermore, we found that STAT3 was a target of miR-93-3p in DLBCL. Rescue assays confirmed that STAT3 mediated the regulation of lncADEI in the immune escape and progression of DLBCL. STAT3 is an important member of the STAT family, and dysregulation of STATs is associated with many cancer types [30, 31]. Li et al. [32] found that the EBV-encoded protein LMP2A upregulated the expression of EHF through phosphorylation of STAT3, and ultimately promoted the growth of gastric cancer cells. Liu et al. [33] reported that TSP1 upregulates PD-L1 expression through the STAT3 pathway in osteosarcoma, thereby achieving immunosuppression. Our results showed that STAT3 binds to the PD-L1 promoter to activate its transcription in DLBCL. Together, lncADEI could regulate STAT3 expression by binding to miR-93-3p, thus further promoting DLBCL progression and immune escape through STAT3 transcriptional upregulation of PD-L1. Binding of PD-L1 to its receptor PD-1 inhibits proliferation of activated T cells has become the main therapeutic mechanism of PD-1/PD-L1 blockade [34]. Here in EBV + DLBCL cells, lncADEI upregulated PD-L1 expression through STAT3, induced more CD8+Tcell exhaustion compared with EBV-DLBCL. These discrepancies could be abrogated by anti-PD-L1 antibody. Together, although potentially oncogenic, lncADEI was a potential target for anti-PD-1 antibody treatment of DLBCL.

Previous studies have pointed out that EBV-DLBCL patients with EBV+ bystander cells have similar clinical features to EBV + DLBCL, shorter survival times, and poorer prognoses than patients without any detectable EBV+ cells [35]. Similarly, we demonstrated that EBV-positive DLBCL promoted the proliferation of surrounding EBV-DLBCL cells. These results indicate that EBV+ and EBV-DLBCL cells can interact with each other. Exosomes are membranous vesicles secreted by cells that are naturally present in body fluids. Exosomes play important roles in cell communication by transferring nucleic acids, lipids, and proteins, with further potential effects on cancer progression, metastasis, immunity, and resistance [36]. Our results showed that EBV + DLBCL secreted more exosomes than EBV-DLBCL. Similarly, Asuka et al. found that EBV infection promoted the release of exosomes from lymphoma cells [37]. After co-culture with EBV + DLBCL exosomes, we found that EBV-DLBCL cells could take up exosomes and the expression level of lncADEI increased. Furthermore, after co-culture with lncADEI-OE exosomes, EBV-DLBCL cell proliferation and clonogenic ability were enhanced and the proportion and cytotoxicity of CD8+ T cells in the tumor microenvironment decreased. These results suggest that lncADEI-rich exosomes secreted by EBV + DLBCL cells can promote EBV-DLBCL progression and immune escape.

As a natural protective carrier of ncRNAs, exosomes can stably transmit signals in normal cells, cancer cells, and their microenvironment [38], indicating the potential role of exosomal lncRNAs as biomarkers in EBV-related DLBCL. We found that lncRNA expression was higher in exosomes from the serum of EBV + DLBCL patients than EBV-DLBCL patients and was associated with worse pathological types and clinical symptoms. However, owing to time limitations, the prognostic value of plasma exosome lncADEI in DLBCL cannot be determined. In the article, the number of case specimens of DLBCL, especially EBV + DLBCL samples, is limited, and it is a single-center study. Gene research based on exosomes requires multi-center, large-sample validation to be more convincing. Furthermore, EBV predominantly exists in DLBCL through either Type II or Type III latency patterns [39]. The EBV + DLBCL cell line we established exhibits Type III latency in which all latent genes are expressed, including EBNA1, EBNA2, EBNA3a, EBNA3b, EBNA3c, LMP1, and LMP2 [40]. Although it highly expresses PD-L1, similar to EBV + DLBCL [41], it does not fully represent the characteristics of EBV + DLBCL patients. Future studies are needed to further elucidate the regulatory mechanisms of exosomal lncADEI in EBV + DLBCL.

## Conclusions

Our study is the first to reveal that the lncRNA ADEI promotes the progression of EBV + DLBCL in vitro and in vivo, and participates in EBV +/− cell communication via exosomes. Mechanistically, lncADEI can regulate STAT3 expression via binding to miR-93-3p in EBV + DLBCL cells to activate PD-L1, leading to the inactivation of CD8+ T cells and contributing to the immune evasion of DLBCL cells (Fig. 6D). These findings suggest a new therapeutic strategy for treating EBV-associated lymphoid malignancies.

## Supplementary information

Figure S1

Figure S2

Supplemental figure legend

Supplemental methods

Supplemental table 1

original WB

## Data Availability

Further information and requests for reagents and resources should be directed to and will be made available by the lead contact Jianzhen Shen upon reasonable request.
