# Supplementary figures and images for: Long noncoding RNA ADEI/miR-93-3p/STAT3 axis promotes Epstein–Barr virus-positive diffuse large B-cell lymphoma progression and immune evasion through regulating the PD-1/PD-L1 checkpoint
Source: Cell Death Dis. 2026 Mar 3;17(1):280. doi: 10.1038/s41419-026-08532-4 (PMC13018472; doi:10.1038/s41419-026-08532-4)

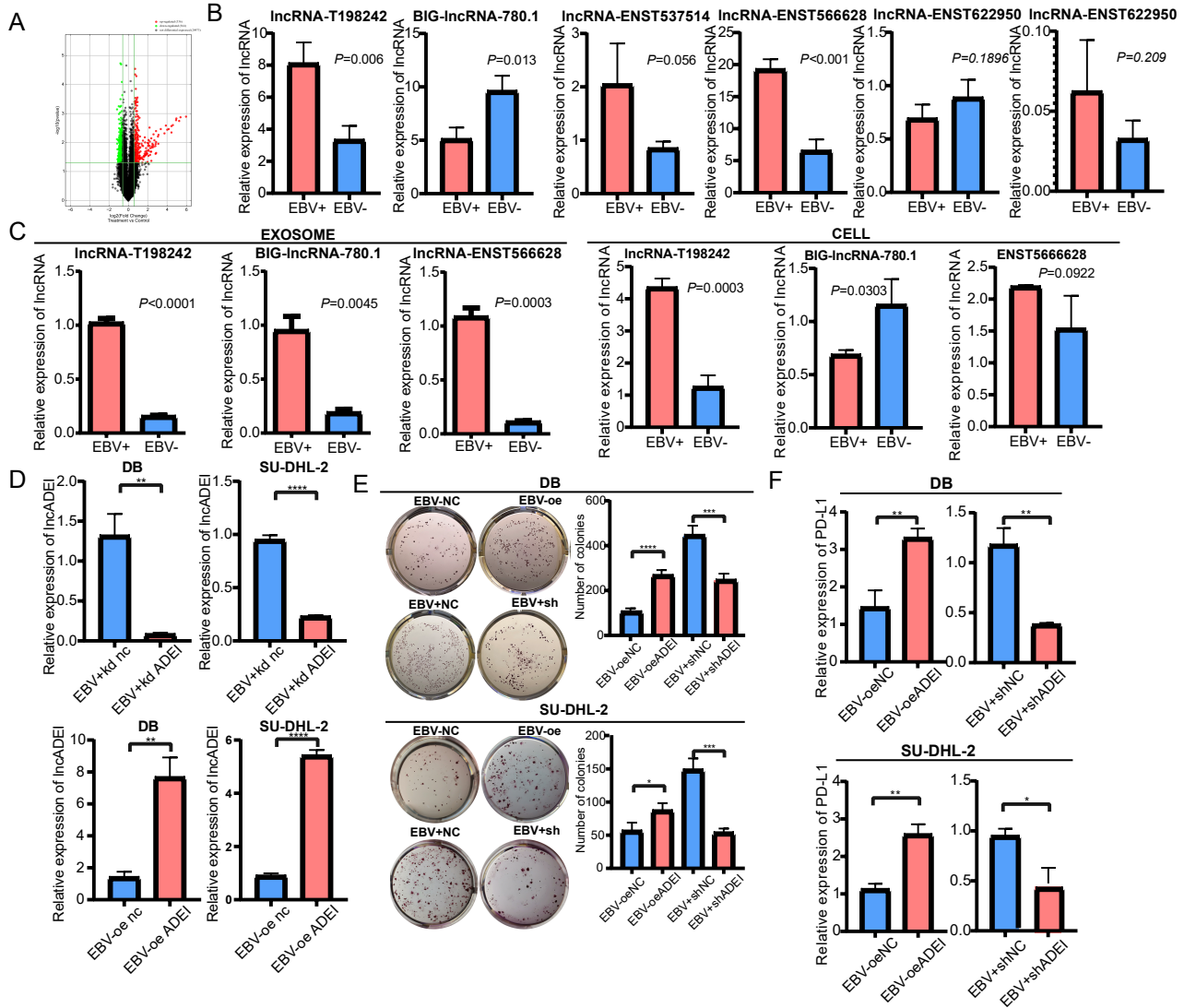

Supplement: Supplementary file 1 — Figure S1 [file 41419_2026_8532_MOESM1_ESM.pdf]

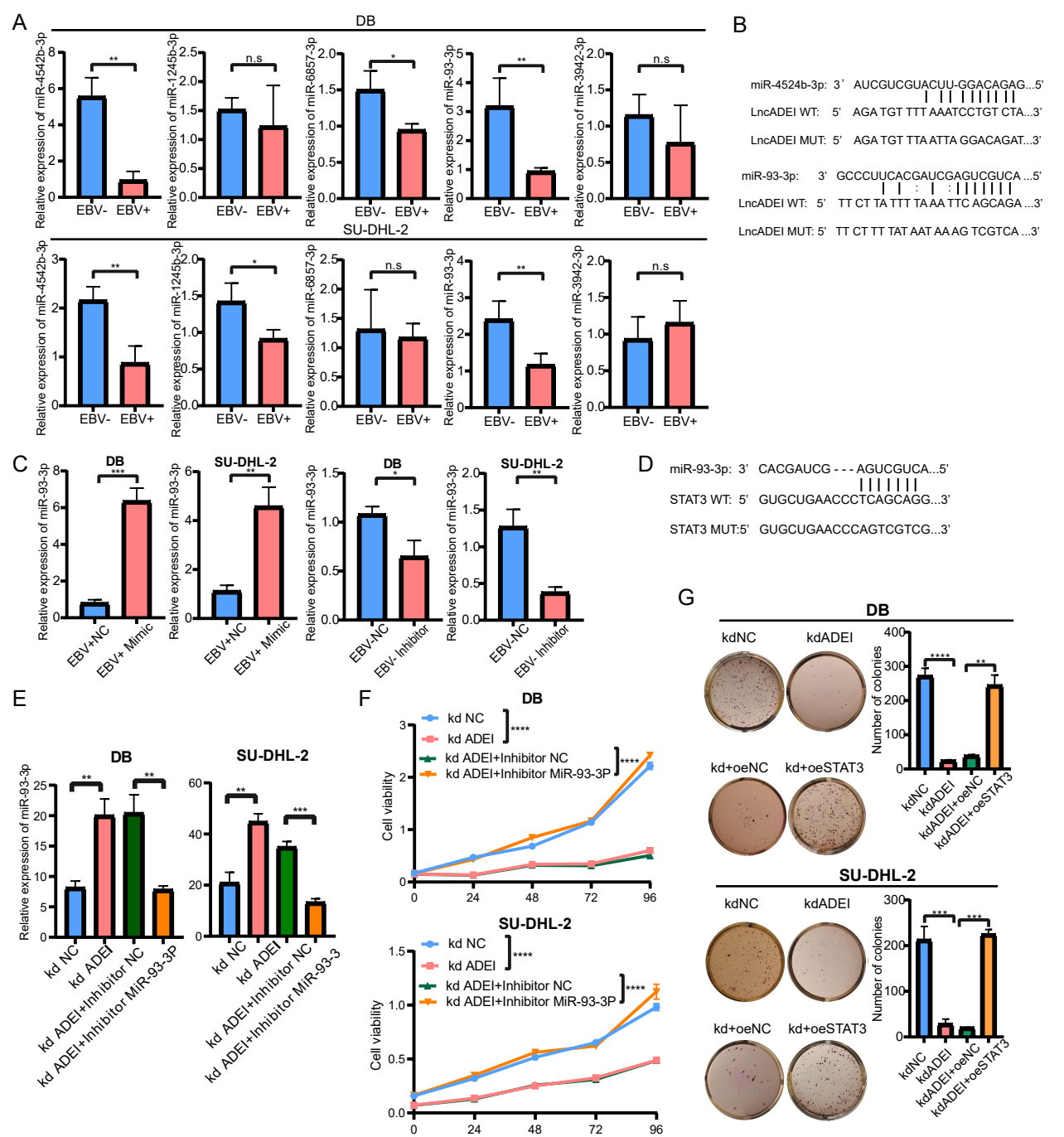

Supplement: Supplementary file 2 — Figure S2 [file 41419_2026_8532_MOESM2_ESM.pdf]

Fig1E

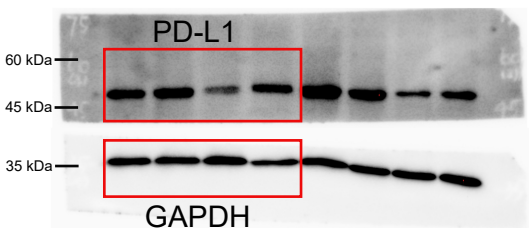

Fig2E

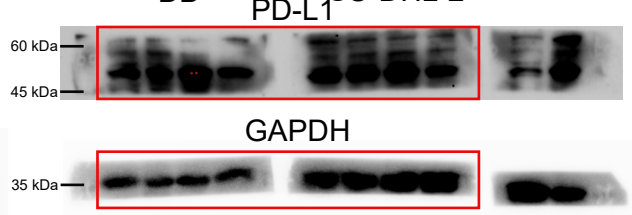

Fig 3F

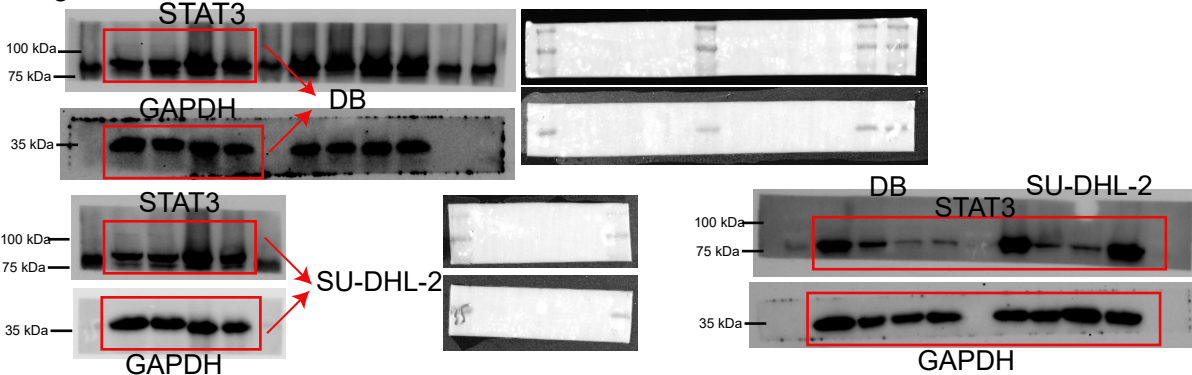

Fig 3I

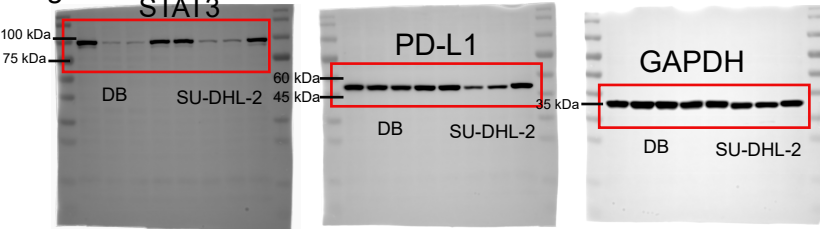

Fig 4B

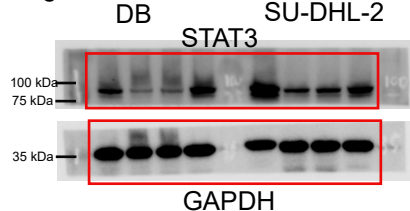

Fig 4F

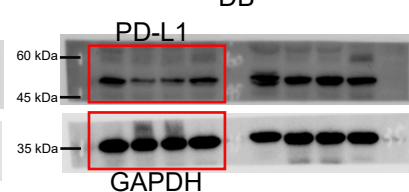

SU-DHL-2

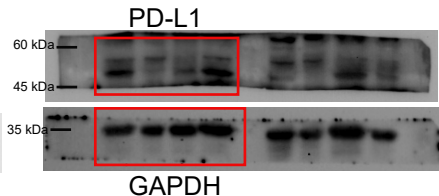

Supplement: Supplementary file 6 — original WB [file 41419_2026_8532_MOESM6_ESM.pdf]
