# Supplementary material for: Long noncoding RNA ADEI/miR-93-3p/STAT3 axis promotes Epstein–Barr virus-positive diffuse large B-cell lymphoma progression and immune evasion through regulating the PD-1/PD-L1 checkpoint
Source: Cell Death Dis. 2026 Mar 3;17(1):280. doi: 10.1038/s41419-026-08532-4 (PMC13018472; doi:10.1038/s41419-026-08532-4)
Supplement: Supplementary file 3 — Supplemental figure legend [file 41419_2026_8532_MOESM3_ESM.docx]

Fig. S1: A: Volcano plot showing the differentially expressed lncRNAs in exosomes of EBV+DB cells compared with EBV-DB. Red and green symbols indicate lncRNAs that were significantly up- and down-regulated, respectively. B: The significantly different lncRNAs of EBV+/- DB exosomes verified in EBV+/-DB cells; 3 of them were differentially expressed. C: The q-PCR analysis was used to verify the expression of different lncRNAs in EBV+/-SU-DHL 2 cells and exosomes. D: The inhibition of lncADEI in lncADEI^sh^EBV+DLBCL and overexpression of lncADEI in lncADEI^oe^EBV-DLBCL cells were confirmed with q-PCR assays. E: The quantification and representative images of transfected cells analyzed by colony formation assays. F: The qRT-PCR analysis shows low expression of PD-L1 in lncADEI^sh^EBV+DLBCL compared with EBV+DLBCL and high expression of PD-L1 in lncADEI^oe^EBV-DLBCL compared with EBV-DLBCL. All data represented mean ± s.d. from three independent experiments. *P < 0.05; **P < 0.01; ***P < 0.001; ****P < 0.0001.

Fig. S2: A: The expression of 5 microRNAs associated with lncADEI in EBV+/-DLBCL cells. B: Interaction sequences of lncADEI for miR-4542b-3p and miR-93-3p binding acquired from bioinformatics prediction and mutated by altering them with complementary sequences. C: The overexpression of miR-93-3p in miR^mimic^EBV+DLBCL cells and inhibition of miR-93-3p in miR^inhibitor^EBV-DLBCL cells were confirmed with q-PCR assays. D: Interaction sequences of STAT3 for miR-93-3p binding acquired from bioinformatics prediction and mutated by altering them with complementary sequences. E: The expression of miR-93-3p in EBV+DLBCL cells after co-transfection with kdADEI and miR-93-3P inhibitor vectors. F: Cell proliferation ability of EBV+DLBCL cells after co-transfection with kdADEI and miR-93-3P inhibitor vectors was detected using CCK-8 assays. G: The quantification and representative images of transfected EBV+DLBCL cells analyzed by colony formation assays (EBV+DLBCL cells were transfected with sh-NC, sh-lncADEI, sh-lncADEI+oe-NC, and sh-lncADEI+oe-STAT3 viruses, respectively).
