# Supplementary material for: Long noncoding RNA ADEI/miR-93-3p/STAT3 axis promotes Epstein–Barr virus-positive diffuse large B-cell lymphoma progression and immune evasion through regulating the PD-1/PD-L1 checkpoint
Source: Cell Death Dis. 2026 Mar 3;17(1):280. doi: 10.1038/s41419-026-08532-4 (PMC13018472; doi:10.1038/s41419-026-08532-4)
Supplement: Supplementary file 4 — Supplemental methods [file 41419_2026_8532_MOESM4_ESM.docx]

**Methods**

**Exosome extraction and identification**

For exosome purification, EBV+/- DLBCL cells were grown in medium containing 10% exosome-depleted fetal bovine serum. Exosomes were collected from the cell culture medium supernatant and serum samples through standard ultracentrifugation steps, as previously described[15]. The morphology and size of the exosomes were observed by transmission electron microscopy (TEM). The concentration, size distribution, and zeta potential of the isolated exosomes were quantified using NanoSight NS300 instrument (Malvern Instruments Ltd. UK) equipped with nanoparticle tracking analysis. CD9 and CD81 proteins were used as markers to identify exosomes using nanoflow cytometry (Beckman Coulter, Pasadena, CA). Exosomes were diluted and incubated with fluorescently-labeled antibodies (CD9, CD81) at 37 ℃ for 30 min. Samples were analyzed by nanoFCM to detect CD9 and CD81 in each group.

**Microarray analysis**

RNA expression profiling was performed using Arraystar Human LncRNA Microarray V5.0. Differentially expressed lncRNAs were identified using fold-change filtering. Hierarchical clustering was performed using Agilent GeneSpring GX software (version 11.5.1).

**RNA extraction, reverse transcription, and quantitative real‑time polymerase chain reaction (qRT-PCR)**

Total RNA was extracted from cells using the TRIzol reagent (Invitrogen, Carlsbad, USA). Reverse transcription RNA was performed using the First Strand cDNA Synthesis Kit (Thermo Fisher Scientific, USA). Relative expression levels were detected using SYBR Green Master (Roche, Switzerland) and Biosystems 7500 Real-Time PCR Systems and calculated using the 2-ΔΔ Ct method. GAPDH and U6 were used as internal controls. The primers used for qRT-PCR analysis are listed in Supplementary Table 1.

**Plasmid construction and transfection**

Specific shRNAs and oeRNAs against lncADEI (sh-lncADEI, oe-lncADEI), sh-NC, and oe-NC were obtained using the pLVshRNA and pCDH-CMV vectors designed by Miaolingbio (Wuhan, China). Plasmids were transfected into HEK-293 T cells to package the lentivirus using the Lipofectamine 3000 reagent. EBV+/- DLBCL cells were infected with the packaged lentivirus and then selected using 2 μg/mL of puromycin (Sigma, USA) for 14 days until lncADEI was stably transfected. MicroRNA and NC mimic/inhibitor were generated by Genechem (Shanghai, China) and transfected with Lipofectamine 3000 reagent (Invitrogen).

**Luciferase report assay**

The sequences of the microRNA-binding site of lncADEI or 3′-UTR of STAT3, as well as their corresponding mutants, were designed, synthesized, and inserted into the luciferase reporter vector pmirGLO (Miaolingbio, China). The luciferase reporter vector and microRNA mimics were transfected into 293T cells using Lipofectamine 3000. The PD-L1 promoter WT/Mut was included in the pGL3-basic vector (Miaolingbio, China) and transfected into 293T cells with oe-STAT3 or oe-NC. Luciferase activity was measured using the Dual-Luciferase Reporter Assay System (E2920, Promega), and a Glomax 96 spectrophotometer was used to detect fluorescence intensity.

**Cytoplasm and nuclear localization**

The cytoplasmic and nuclear fractions were separated and isolated from the cells using a PARIS kit (Ambion, Austin, TX, USA) according to the manufacturer’s protocol. Cytoplasmic and nuclear RNAs were reverse-transcribed to cDNAs and detected by q-PCR.

**Cell proliferation and apoptosis**

The B lymphoma cells were seeded in 24-well plates. Following incubation for 0, 24, 72, or 96 hours, Cell Counting Kit (CCK)-8 (Dojindo, Japan) was added, and the absorbance of each well was measured at 450 nm by spectrophotometry. An APC Annexin V Apoptosis Detection kit (Biolegend, San Diego, USA) was used to analyze the cells, following the manufacturer’s instructions, and a BD Accuri C6 flow cytometer was used to analyze the samples.

**Exosome uptake assay**

EBV+ exosomes were extracted from the cell supernatant and stained with PKH26 (Umibio, Shanghai,China) according to the manufacturer’s protocol. Stained exosomes were added to the EBV-negative DLBCL (EBV-DLBCL) cells and incubated for 48 hours. After immobilization and DAPI staining, cells were imaged using a laser confocal microscope.

**In vitro co-culture system and flow cytometry**

In vitro co-culture was performed in a Transwell cell incubator (Millipore Corporation, USA). In the co-culture system, lymphoma cells were placed in the upper cavity, and immune cells were placed in the lower cavity, with both cells in direct contact. Immune cells were mononuclear cells (including lymphocytes and monocytes) isolated from the peripheral blood of healthy volunteers by density gradient centrifugation using Ficoll. CD8+ T cells were surface stained with an anti-CD8 antibody (555369, BD) and sorted using the FACSAria Cell Sorter. PD-1 expression in CD8+ T cell was assessed using the anti-PD-1 antibody (367404, Biolegend). INF- γ + CD8+ T cell was assessed using the anti- INF- γ antibody (559326, Biolegend) after cell membrane disruption. The percentage of CD8+ T cells or INF- γ in CD8+ T cells, PD-1 expression on CD8+ T cells were detected using flow cytometry.

**In vivo tumorigenesis assay**

All the animal experiments were conducted in accordance with approved protocol by the Animal Ethics Committee of Clinical Oncology School of Fujian Medical University. A complete randomization grouping method is employed, where animals are assigned to the experimental group and the control group using a random number table to ensure the initial conditions of each group are balanced. Nude mice (4 weeks old) from Shanghai Laboratory Animal Center were randomly injected with stably transfected DLBCL cells into the right flank. The investigators were blind to the treatment groups during the experiment and outcome assessment. Tumor volumes were calculated as 0.5×a (length)×b (width) ^2^.
