# Supplementary material for: Long noncoding RNA ADEI/miR-93-3p/STAT3 axis promotes Epstein–Barr virus-positive diffuse large B-cell lymphoma progression and immune evasion through regulating the PD-1/PD-L1 checkpoint
Source: Cell Death Dis. 2026 Mar 3;17(1):280. doi: 10.1038/s41419-026-08532-4 (PMC13018472; doi:10.1038/s41419-026-08532-4)
Supplement: Supplementary file 5 — Supplemental table 1 [file 41419_2026_8532_MOESM5_ESM.docx]

| Gene | Primer | 5’-3’ |
| --- | --- | --- |
| EBER2 | Forward | AGGACAGCCGTTGCCCTAGTGGTTTCG |
|  | Reverse | AAAAACAGCGGACAAGCCGAATACC |
| EBNA1 | Forward | GATGAGCGTTTGGGAGAGCTGATTCTGCA |
|  | Reverse | TCCTCGTCCATGGTTATCAC |
| EBNA2 | Forward | GCTGCTACGCATTAGAGACC |
|  | Reverse | TCCTGGTAGGGATTCGAGGG |
| LMP1 | Forward | TCCTCCTCTTGGCGCTACTG |
|  | Reverse | TCATCACTGTGTCGTTGTCC |
| PD-L1 | Forward | CGGAGTATGCCACCATTGTC |
|  | Reverse | CCAAGAGCAGTGTCCATCCT |
| ENST00000537514 | Forward | TAGGGGCACAAAGTGGTCA |
|  | Reverse | CACAGCAGAGTATTCGGTGG |
| T198242 | Forward | CTTCCACCCACATTCCAAA |
|  | Reverse | TGAATCTCATCAAGCCCCTC |
| ENST00000622950 | Forward | CAAATGAAGAAATGGAAGTCAA |
|  | Reverse | GCTGAACTTGGGAGCCTTAA |
| ENST00000566628 | Forward | TTTTAAGTGCTGTTTGTGGGGT |
|  | Reverse | GCTCCTCCTCAGAATGGCAACT |
| BIG-lncRNA-780.1 | Forward | GAAAGGTTCAATTCTGTAAGATG |
|  | Reverse | TTCTTTTGATTGTGCAGTTTTG |
| ENST00000623638 | Forward | TCTTTCCCATCCATGTCTGTC |
|  | Reverse | TCTAGTTTTCCTTATGGGGTGT |
| hsa-miR-4524b-3p | Forward | CCGGAGACAGGTTCATGCTGCTA |
| hsa-miR-1245b-5p | Forward | CCGCGCTAGGCCTTTAGATCACTTAAA |
| hsa-miR-6857-3p | Forward | CTGACTGAGCTTCTCCCCACAG |
| hsa-miR-93-3p | Forward | ATACTGCTGAGCTAGCACTTCCCG |
| hsa-miR-3942-3p | Forward | CCGCGCGTTTCAGATAACAGTATTACAT |
| U6 | Forward | CTCGCTTCGGCAGCACA |
|  | Reverse | AACGCTTCACGAATTTGCGT |
| STAT3 | Forward | TTGCCAGTTGTGGTGATC |
|  | Reverse | AGACCCAGAAGGAGAAGC |
